# Book Reviews

**Published:** 1896-02

**Authors:** 


### *BOOK REVIEWS.*

An American Text-Book of Obstetrics. For Practitioners and Students. By Jas. C. Cameron, M.D., Edward P. Davis, M.D., Robert L. Dickinson, M.D., Barton C. Hirst, M.D., Howard A. Kelley, M.D., Theophilus Parvin, M.D., George A. Piersol, M.D., and others. Richard C. Norris, M.D., Editor. Published by W. B. Saunders, 925 Walnut street, Philadelphia. Price, cloth, $7.00.

Many excellent treatises upon obstetrics have appeared during the past few years, and hence it would have seemed that there was no "long-felt want" to be filled by a new one. But a perusal of the volume before us will lead to a different conclusion. We can safely say, without fear of contradiction, that this is by all odds the best work on the subject in the English language at the present day. For completeness, for accuracy of detail, for clearness of statement, and for practical usefulness, it stands without a peer. It is not intended for a manual or a handbook. It is large, because the subject is a large one. It argues, it theorizes, because many points in obstetrics are still *sub judice.* But its arguments and theories do not obscure its plain statements of facts, where facts are known. It presents, thoroughly and completely, the present aspect of obstetric science and art from the American standpoint.

Not the least valuable feature of the book are the illustrations, of which there are nearly nine hundred, many being half-tone reproductions of photographs. So finely executed is this department of the work, that the volume might almost be regarded as an *édition de luxe.* There are no reproductions of antiquated plates, inserted to fill space, but all are new and up-to-date, and are valuable aids to an understanding of the text.

Altogether the work is the most valuable addition to obstetric literature that America has yet produced, and the editor, his collaborators, and the publisher deserve the thanks, as well as the congratulations, of the profession. V. O. H.

THE DEFORMITIES OF THE HUMAN FOOT, WITH THEIR TREATMENT. By W. J. Walsham, M.B., F.R.C.S., Senior Assistant Surgeon, Surgeon in charge of the Orthopedic Department and Lecturer in Anatomy, St. Bartholomew's Hospital; and William Kent Hughes, M.B., London, Orthopedic Surgeon, St. Vincent's Hospital. Extra Muslin, Octavo, $4.50. New York: William Wood & Company.

This volume contains the results of the authors' thirteen years' experience in the Orthopedic Department of St. Bartholomew's Hospital, and as the book is written from the standpoint of the general surgeon rather than the orthopedic specialist, it is eminently practical in character. The authors not only present a record of their experience with the eight hundred patients treated annually in the Orthopedic Department, but have also freely availed themselves of the labors of other writers. The anatomy and mechanism of the normal foot are discussed at some length and well illustrated. The deformities of the foot, including club foot and flat foot, with an extensive review of their anatomy, causation, and treatment, constitute the great bulk of the work, and contain many original illustrations and much that is of great practical value to physicians and surgeons who are called upon to treat these deformities. Nor are the minor deformities of the feet, as hallux valgus, varus, and dolorosa, hammer-toe, lateral deviation of small toe, deficiency of the toes, hypertrophy of the toes, etc., neglected. The volume is well written and freely illustrated. F. W. M.

---

SYSTEM OF SURGERY. By American Authors. Edited by Frederic S. Dennis, M.D., Professor of the Principles and Practice of Surgery, Bellevue Hospital Medical College, Visiting Surgeon to Bellevue Hospital, etc. Assisted by John S. Billings, M.D., Deputy Surgeon-General, U. S. A. Volume III. Profusely Illustrated. Published by Lea Bros. & Co., 706–710 Sansom St., Philadelphia. Price $6.00.

The third volume of this splendid system of surgery deals with the Surgery of the Larynx and Trachea, by Dr. D. Bryson Delavan, of New York; Surgery of the Mouth and Tongue, by Dr.

H. H. Mudd, of St. Louis; Diseases of the Salivary Glands, by Dr. Chas. B. Porter, of Boston; Surgery of the Neck, by Dr. Willard Parker, of New York; Surgical Diseases and Injuries of the Chest, by the Editor, Dr. Dennis; Diseases of the Eye, by Dr. G. E. de Schweinitz, Philadelphia; Operative Surgery of the Eye, by Drs. H. D. Noyes and J. E. Weeks, New York; Surgery of the Ear, by Dr. Gorham Bacon, New York; Surgical Diseases of the Jaws and Teeth, by Dr. L. McLane Tiffany, Baltimore; Surgical Diseases of the Skin, by Dr. W. A. Hardaway, St. Louis; Surgery of the Genito-Urinary System, by Drs. J. William White and W. H. Furness, Philadelphia; and Syphilis, by Dr. R. W. Taylor, New York.

The subjects discussed in this volume are presented in such a manner as to be both complete and practical, and so far as pathology and modes of treatment go, all of them are brought strictly up to present time. The section on the Genito-Urinary System is the most extensive, embracing about 450 pages, and almost a complete work in itself. The section on Syphilis is practically a condensation of Dr. Taylor's book on this subject, than which there is nothing better. One more volume will complete this system.

---

ANNUAL OF THE UNIVERSAL MEDICAL SCIENCES. A Yearly Report of the Progress of the General Sanitary Sciences. Edited by Chas. E. Sajous, M.D., and Seventy Associate Editors, 1895. The F. A. Davis Company, Publishers, 1916 Cherry St., Philadelphia.

The successive issues of this useful work have been reviewed in these pages every year, and doubtless many of our readers annually add the volumes to their libraries; so that they are thoroughly familiar with its character and scope. The present set of five volumes is uniform with those previously issued, giving evidence of the same care and discrimination in the selection of matter which have been observed in preceding volumes. Some new names are noticed in the list of associate editors. Every subject relating to medicine, hygiene, and sanitary science has been well covered by the editors, and their combined work is an admirable digest of all medical literature important enough to find mention in a work of this sort.

This eighth issue of the *Annual* is fully the equal of its predecessors in point of merit, and equally deserving of a place in the library. We notice the misspelling of several proper names. Subsequent issues should be more careful in this.

---

OUTLINES OF MATERIA MEDICA AND PHARMACOLOGY. A Text-Book for Students. By H. M. Bracken, M.D., Professor of Materia Medica and Therapeutics, University of Minnesota. Published by P. Blakiston, Son & Co., 1012 Walnut St., Philadelphia. Price $2.75.

Any new text-book should have as a *raison d'être* some particular feature of usefulness or merit not possessed by its predecessors. By this standard we do not see what has been gained by the present work. It may have some value to the students to whom the author lectures on materia medica and therapeutics, but for a general text-book on this subject it is far behind many others which we have been using for several years. In the arrangement of his materia medica, both mineral and vegetable, the author adopts neither the physiological nor the alphabetical classification, the drugs being thrown together without any apparent attempt at order and system. The therapeutic uses of drugs receive very little attention. As said before, we do not see what is gained by this work.

---

PRINCIPLES OF SURGERY. By N. Senn, M.D., PH.D., LL.D., Professor of Practice of Surgery and Clinical Surgery in Rush Medical College, Chicago; Attending Surgeon to the Presbyterian Hospital, etc., etc. Second Edition. Thoroughly revised. Illustrated with 178 Wood Engravings and Five (5) Colored Plates. Extra Cloth, $4.50; Sheep or Half-Russia, $5.50. Philadelphia. The F. A. Davis Co., Publishers, 1914 and 1916 Cherry street.

The first edition of this work and its author are too well known to require an extended introduction of the present edition. This second edition is full and thorough, containing descriptions of all the conditions which are to be dealt with in considering the Principles of Surgery.

The book is up-to-date. Besides the simple principles many points in their practical application and in the actual practice of surgery are given and illustrated. This feature is to be commended as relieving the subject of the dryness of generalities by showing the interest of actual practice. The histological drawings are excellent. It is a valuable text-book. M. B. H.

---

A MANUAL OF THE PRACTICE OF MEDICINE. By George R. Lockwood, M.D., Professor of Practice in the Woman's Medical College, New York, Attending Physician to the City Hospital, etc. Published by W. B. Saunders, 925 Walnut St., Philadelphia. Price $2.50.

The aim of this Manual is to "present the essential facts and principles of the practice of medicine in a concise and available form." The author has adopted the classification of Osler, and has succeeded very well in making a practical and useful working manual of practice. The book comprises 925 pages, and contains seventy-five illustrations in the text and twenty-two full page colored plates. The volume will be especially serviceable and convenient for medical students.

---

A TEXT-BOOK OF PHYSIOLOGY. By M. Foster, M.A., M.D., LL.D., F.R.S., Professor of Physiology in the University of Cambridge, Fellow of Trinity College, Cambridge. Sixth American Edition, thoroughly revised, with notes, additions, and two hundred and fifty illustrations. Lea Brothers & Co., 706 Sansom St., Philadelphia.

Previous editions of this excellent, standard work have been reviewed in these pages. This is a new edition which the American publishers have brought out with such emendations and changes as seemed warranted by the needs of the American student and practitioner. Needless to say, the book meets all the requirements of those desiring a complete text-book of physiology. M. B. H.

---

LIPPINCOTT'S MAGAZINE FOR FEBRUARY, 1896.

The complete novel in the February issue of Lippincott's is "Ground-swells," by the well-known writer, Mrs. Jeannette H.

Walworth. It is a tale of rather unusual length (for the magazine), readable, lively, and "up-to-date." The scene is in New York city, and the heroine is, or tries to be, a New Woman.

"Fifteen," by Marjorie Richardson, is the tale of a high-minded cash-boy, supposed to be told by one of his comrades in the dry-goods store.

Dr. Harvey B. Bashore gives an interesting epitome of the furthest researches of geology in a rapid sketch of "The First Days of the World." "The Aërial Monasteries of Greece" are de-described by Charles Robinson.

James Knapp Reeve writes of "What Men Drink." E. S. F. gives some account of "Domestic Service on the Pacific Slope" and the difficulties thereof.

"The Child and his Fictions" is a pleasant and suggestive paper by Elizabeth Ferguson Seat. Frederic M. Bird points out certain "Paralyzers of Style," some of which are intended to have a precisely opposite effect, while some are the result of mere carelessness.

The poetry of the number is by Joseph Wharton, Charles G. D. Roberts, and Clinton Scollard.

---

THE COSMOPOLITAN.

No one ever thought of introducing so expensive a feature as lithographic color work in the days when the leading magazines sold for $4.00 a year and 35 cents a copy. But times change, and the magazines change with them. It has remained for the *Cosmopolitan*, sold at one dollar a year, to put in an expensive lithographic plant capable of printing 320,000 pages per day (one color). The January issue presents, as a frontispiece, a water-color drawing by Eric Pape, illustrating the last story by Robert Louis Stevenson, which has probably never been excelled, even in the pages of the finest dollar French periodicals. The *Cosmopolitan* is also changed, a drawing, of page length, by the famous Paris artist, Rossi, in lithographic colors on white paper, takes the place of the manilla back with its red stripes. Hereafter the cover is to be a fresh surprise each month.

